# Prevalence, predictors and management of left atrial appendage thrombogenic milieu in atrial fibrillation with low thromboembolic risk

**DOI:** 10.1186/s12959-023-00478-3

**Published:** 2023-03-30

**Authors:** Yu Qiao, Zhen Zhao, Xiang Cai, Yulong Guo, Ke Liu, Jinrui Guo, Tao Guo, Guodong Niu

**Affiliations:** 1 Department of Cardiac Arrhythmia, Fuwai Yunnan Cardiovascular Hospital, Kunming Medical University, Kunming, Yunnan, 650032 People’s Republic of China; 2 State Key Laboratory of Cardiovascular Disease, Cardiac Arrhythmia Center, Fuwai Hospital, National Center for Cardiovascular Diseases, Chinese Academy of Medical Sciences, Peking Union Medical College, Beijing, 100037 People’s Republic of China

**Keywords:** Atrial fibrillation, Left atrial appendage, Transesophageal echocardiography, Thromboembolism, Anticoagulation

## Abstract

**Background:**

The present study aimed to investigate the prevalence, predictors, and management of left atrial appendage (LAA) thrombogenic milieu (TM) identified with transesophageal echocardiography (TEE) in non-valvular atrial fibrillation (NVAF) patients with low to moderate thromboembolic (TE) risk.

**Methods:**

We retrospectively analyzed the baseline clinical data and TEE findings in 391 NVAF patients (54.7 ± 8.9 years, 69.1% male) with low to moderate TE risk according to the CHA_2_DS_2_-VASc score. LAA TM was defined as LAA thrombus (LAAT), sludge or spontaneous echo contrast (SEC). Management of LAA TM was at the discretion of the treating physician.

**Results:**

A total of 43 patients (11.0%) were detected with LAA TM, including 5 with LAAT (11.6%), 4 with LAAT + Sect. (9.3%), 3 with sludge (7.0%), and 31 with Sect. (72.1%). In multivariate model, non-paroxysmal AF (OR 3.121; 95% CI 1.205–8.083, p = 0.019), and a larger left atrial diameter (LAD) (OR 1.134; 95% CI 1.060–1.213, p < 0.001) were significantly associated with the presence of LAA TM. All LAATs or sludges effectively resolved after mean duration of 117.5 ± 20.0 days for oral anticoagulant (OAC) medication. TE events occurred in 3 patients (18.8%) among those discontinuing OAC over a mean follow-up of 26.2 ± 8.8 months, while no TE events occurred in patients with continuous OAC.

**Conclusions:**

LAA TM could be identified in 11.0% in NVAF patients with low to moderate TE risk, especially in those with non-paroxysmal AF and enlarged LAD. Short-term OAC medication could effectively resolve the LAAT or sludge.

## Introduction

Atrial fibrillation (AF) is associated with increased risk of death and cardiovascular events, especially thromboembolic (TE) events [1, 2]. Left atrial appendage (LAA) is shown to play a major role in atrial thrombosis and subsequent TE events in non-valvular AF (NVAF) [3–5]. According to current guidelines, the CHA_2_DS_2_-VASc scoring system (congestive heart failure, hypertension, age ≥ 75 years, diabetes mellitus, prior stroke or transient ischemic attack or thromboembolism, vascular disease, age 65 to 74 years, female sex) is recommended in NVAF patients to identify those with high TE risk (≥ 2 points in male or ≥ 3 points in female), who could largely benefit from anticoagulation [6]. However, the real-world TE risk of patients with low to moderate TE risk determined by CHA_2_DS_2_-VASc score has not been completely investigated. Consequently, the indication for long-term anticoagulation in patients with low to moderate TE risk has not been well-established.

Transesophageal echocardiography (TEE) has been widely used to detect the LAA thrombus (LAAT) formation [7]. Recent studies have shown that the presence of LAA thrombogenic milieu (TM), including LAAT, sludge and spontaneous echo contrast (SEC), could serve as a high risk marker for TE events [8–10]. Therefore, the present study aimed to investigate the prevalence, risk factors, and management of LAA TM identified with TEE in NVAF patients with low to moderate TE risk according to CHA_2_DS_2_-VASc score.

## Methods

### Study population

**Fig. 1:**
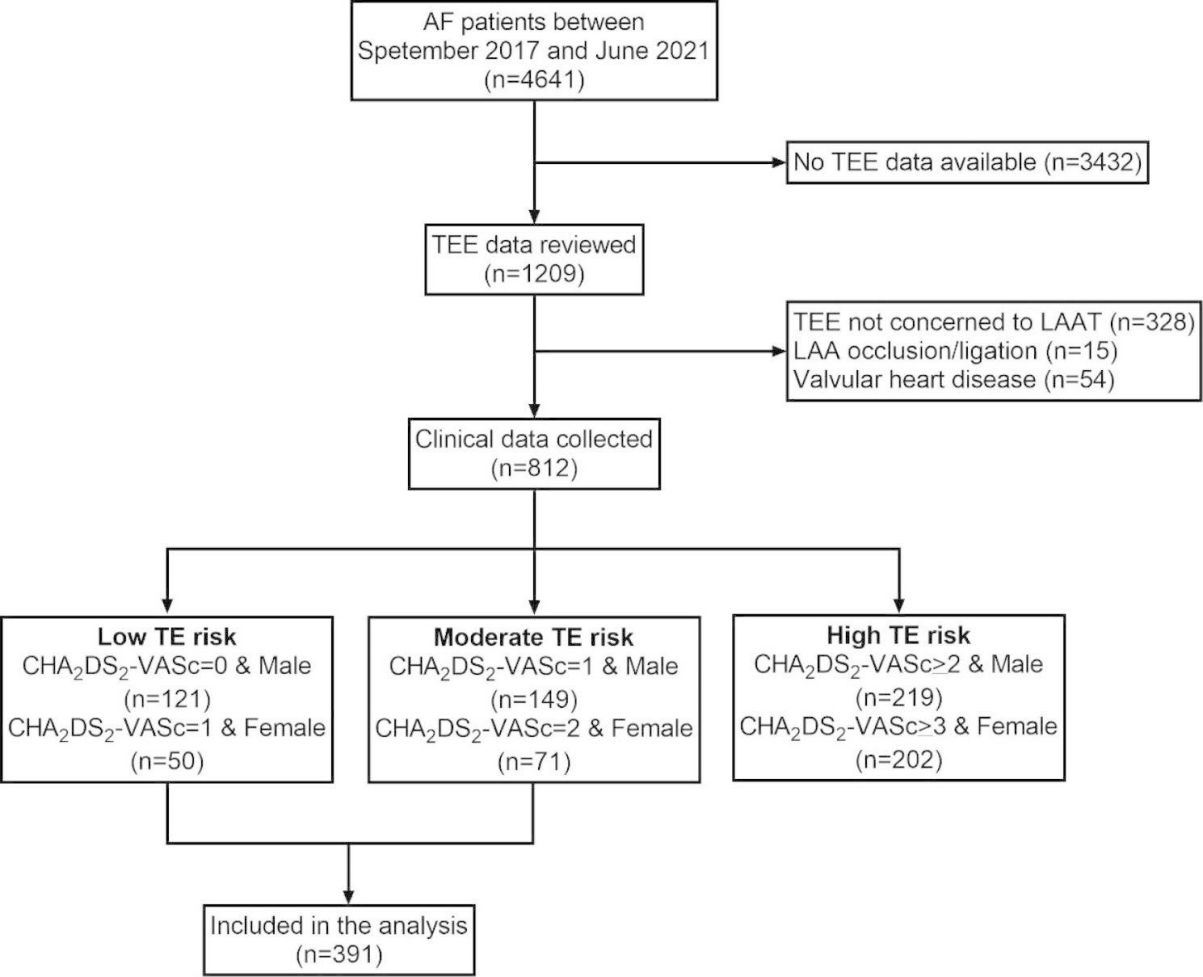
Flow chart of the study design. AF, atrial fibrillation; TEE, transthoracic echocardiography; LAA, left atrial appendage; LAAT, left atrial appendage thrombosis; TE, thromboembolism

In the present retrospective single-center observational study, we screened all in-hospital patients who were diagnosed with AF in our institution between September 2017 and June 2021, among whom, TEE data were available in 1,209 patients. The exclusion criteria were: (1) TEE not relevant to LAA; (2) history of LAA occlusion or ligation; (3) valvular heart disease. After the screening process, the demographic and medical data of the remaining 812 patients were collected. According to CHA_2_DS_2_-VASc score, the patients were further stratified into low (CHA_2_DS_2_-VASc = 0 in male or CHA_2_DS_2_-VASc = 1 in female), moderate (CHA_2_DS_2_-VASc = 1 in male or CHA_2_DS_2_-VASc = 2 in female) and high (CHA_2_DS_2_-VASc ≥ 2 in male or CHA_2_DS_2_-VASc ≥ 3 in female) TE risk groups. Finally, a total of 391 patients with low to moderate TE risk were included in the present study. (Fig. 1) The study protocol was reviewed and approved by the institutional review board. The study complies with the Declaration of Helsinki.

### Echocardiographic studies

Transthoracic echocardiography (TTE) data using a standard two-dimensional and Doppler echocardiography with color flow mapping were reviewed. In case of patients with multiple TTEs, the study from the same hospitalization with TEE was used for data collection and subsequent analysis. Left ventricular ejection fraction (LVEF) was calculated according to the Simpson’s biplane method. Mitral regurgitation (MR) was evaluated according to current recommendations using an integrative approach that includes qualitative, semiquantitative, and quantitative data and classified into four grades: none, mild, moderate and severe.

**Fig. 2:**
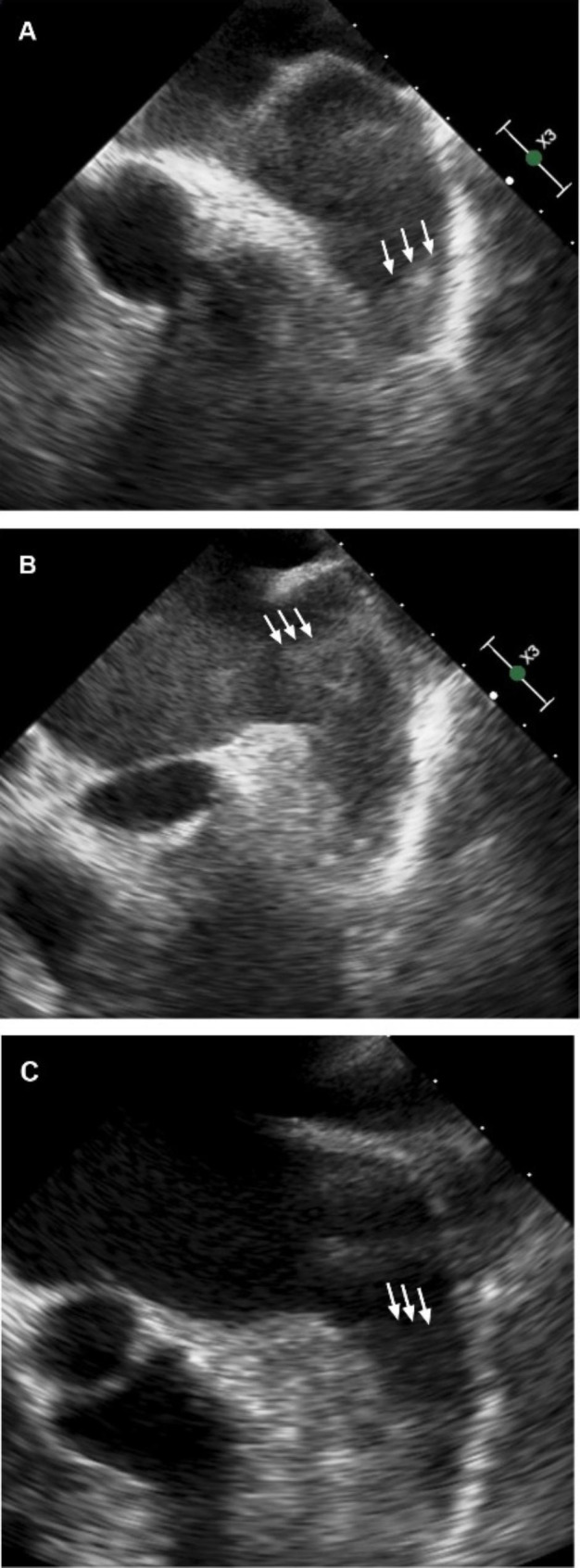
The example of left atrial appendage thrombosis (A), left atrial appendage sludge (B), and left atrial appendage spontaneous echo contrast (C)

TEE was performed after standard clinical preparation with a 5.0-mHZ, 128-element, multiplane probe (Phillips) by experienced echocardiographers who were blinded to the clinical histories of the patients. LAA was scanned in multiple mid-esophageal imaging planes from 0° to 180° to optimize the visualization of the entire LAA. LAAT was defined as a localized echo-dense intracardiac mass distinct to the LAA endocardium and pectinate muscles, which was present in > 1 imaging plane [11] (Fig. 2A). LAA sludge was defined as a viscid gelatinous, precipitous echodensity within the LAA, which could be continuously seen throughout the cardiac cycle without a discrete organized mass [9] (Fig. 2B). LAA SEC was defined as characteristic dynamic swirling echoes within the LAA cavity with optimal gain setting [10] (Fig. 2C).

To evaluate the inter- and intra-observer concordance in assessing LAA TM, a randomly selected group of 20 patients from our database, including LAAT (5), sludge (5), Sect. (5), and normal control (5), was and analyzed by the 2 same observers in a blinded manner at different occasions and time (4 weeks apart), as previously reported [12].

### Management of LAA TM

All patients with LAAT or sludge received oral anticoagulant (OAC) for at least 3 months. The type of OAC was at the discretion of the treating physician. For anticoagulation with a Vitamin K antagonist (VKA), the target international normalized ratio (INR) was 2.0 to 3.0, and time in therapeutic range (TTR) was calculated. A staged TEE was performed 3–4 months after discharge to demonstrate if LAAT or sludge resolved. The treatment of patients with LAA SEC was left to the physician’s discretion and patient’s choice. The clinical data of follow-up period were retrospectively collected in the medical system or through telephone visit.

### Statistical analysis

Continuous variables were described as the mean ± standard deviation for normally distributed data and median (25–75% quartile) for non-normally distributed data. Comparisons between groups were performed with Student t test (normally distributed data) or Kruskal–Wallis test (non‐normally distributed data). Categorical variables were described as counts (percentage) and compared by chi‐square analysis. Kappa statistics were calculated to evaluate the inter- and intra-observer concordance in identification of LAA TM. Binominal logistic regression was used to calculate the odds ratio (OR) and 95% confidence interval (CI) for the presence of LAA TM. Variables selected for testing in the multivariate analysis were those with P < 0.05 in the univariate model. A receiver operating characteristic (ROC) analysis was used to determine the cut-off value of left atrial diameter (LAD) for predicting the presence of LAA TM. All tests were two-tailed, and a statistical significance was established at P < 0.05. All analyses were performed using SPSS software (version 22.0; SPSS, Inc.).

## Results

### Baseline characteristics of the study population

A total of 391 patients with NVAF were included in the study. Mean age was 54.7 ± 8.9 years, and 270 (69.1%) were male. The medium CHA_2_DS_2_-VASc score was 1.0 (0–1.0). The number of patients with CHA_2_DS_2_-VASc score of 0, 1, 2 was 121 (30.9%), 199 (50.9%), and 71 (18.2%), respectively.

**Fig. 3:**
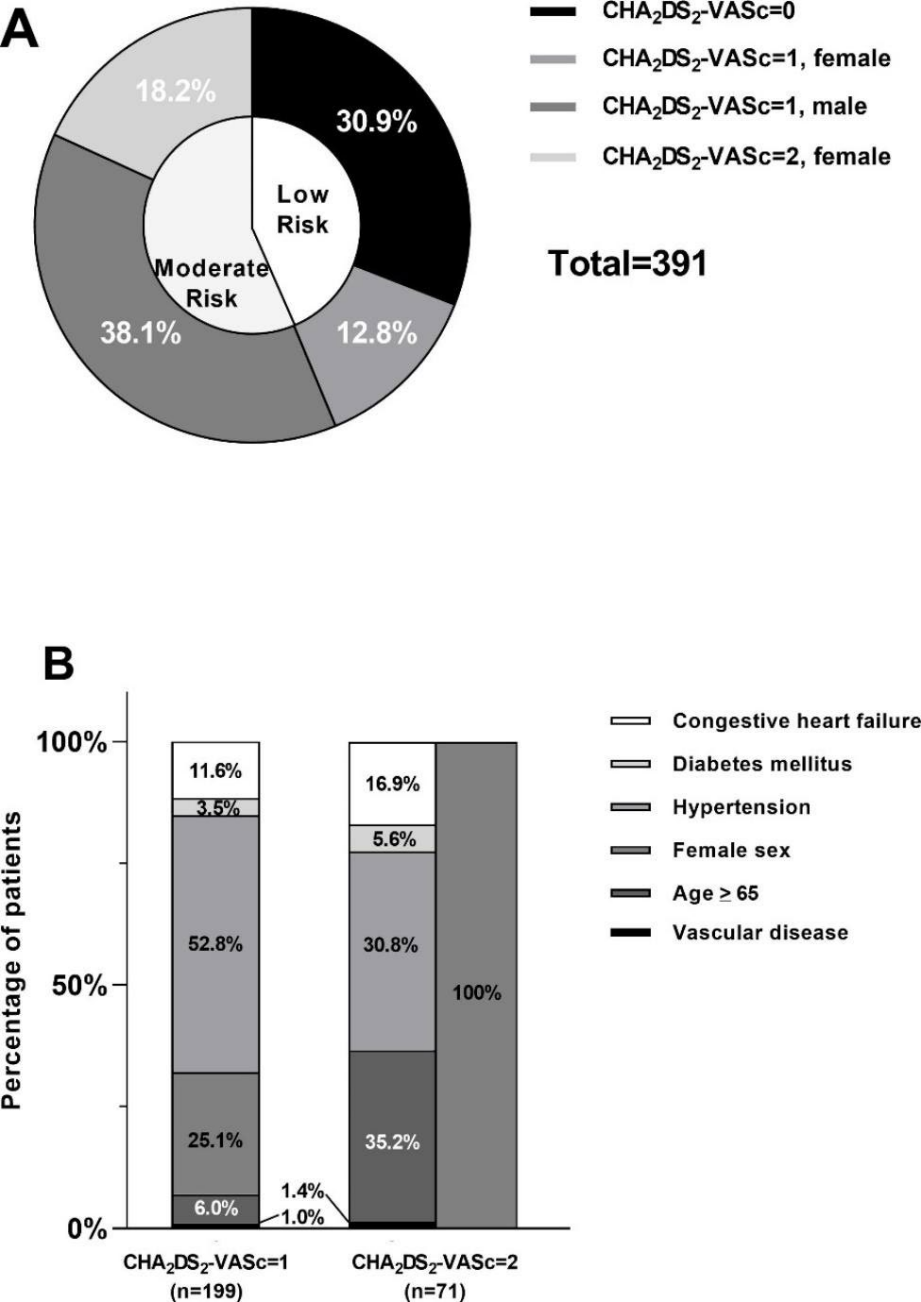
The thromboembolic (TE) risk of the study population. A, TE risk stratification according to the CHA_2_DS_2_-VASc score; B, prevalence of different risk factors in patients with CHA_2_DS_2_-VASc score of 1 and 2

Figure 3 shows the TE risk of the whole study population. According to the CHA_2_DS_2_-VASc score, 171 (43.7%) patients were stratified to low TE risk group, while the remaining 220 (56.3%) patients were at moderate TE risk.

### Inter- and intra-observer concordance

The inter- and intra-observer concordance in identification of different status of LAA TM was both very high, with the overall kappa of 0.92 and 1.00, respectively.

### Prevalence and characteristics of patients with LAA TM

During TEE examination, 199 patients were in normal sinus rhythm. LAA TM was detected in 43 patients (11.0%) in TEE. The baseline characteristics of the patients with and without LAA TM are shown in Table 1. Patients with LAA TM were older (p = 0.025), had a higher prevalence of non-paroxysmal AF (p < 0.001), congestive heart failure (p < 0.001), and had higher CHA_2_DS_2_-VASc scores (p = 0.022), fibrinogen (p = 0.042), N-terminal pro-B type natriuretic peptide (NT-ProBNP) (p < 0.001), and serum uric acid levels (p = 0.033). In transthoracic echocardiography (TTE), patients with LAA TM had significantly larger LAD (p < 0.001), LVEDD (p < 0.001), lower LVEF (p < 0.001) and a higher prevalence of MR (p < 0.001).

More details on patients with LAA TM are shown in Table 2. The number of patients who were detected with LAAT, LAA sludge, LAA SEC was 9, 3, 35, respectively. Notably, 4 patients showed both LAAT and SEC in TEE.

**Table 1:** Baseline characteristics of the study population

Variables	LAA TM		Total n = 391	P value
	Absent n = 348	Present n = 43		
Demographic characteristics				
Age, years old	54.4 ± 9.0	57.6 ± 6.9	54.7 ± 8.9	0.025
Male sex, n (%)	241 (69.3)	29 (67.4)	270 (69.1)	0.809
BMI, kg/m^2^	24.4 ± 3.2	24.8 ± 3.5	24.5 ± 3.3	0.517
Clinical characteristics				
Non-paroxysmal AF, n (%)	111 (31.9)	35 (81.4)	146 (37.3)	< 0.001
Hypertension, n (%)	119 (34.2)	15 (34.9)	134 (34.3)	0.929
Diabetes mellitus, n (%)	8 (2.3)	3 (7.0)	11 (2.8)	0.207
Congestive heart failure, n (%)	23 (6.6)	12 (27.9)	35 (9.0)	< 0.001
Coronary artery disease, n (%)	22 (6.3)	4 (9.3)	26 (6.6)	0.678
CHA_2_DS_2_-VASc score	1.0 (0, 1.0)	1.0 (1.0, 2.0)	1.0 (0, 1.0)	0.022
Laboratory findings				
Hematocrit, %	48.4 ± 8.4	51.1 ± 9.1	48.7 ± 8.5	0.066
Platelet, 10^9^/L	184.5 ± 46.7	179.0 ± 48.7	183.9 ± 46.9	0.484
D-dimer	0.06 (0.03, 0.1)	0.06 (0.04, 0.12)	0.06 (0.03, 0.1)	0.358
Fibrinogen,	2.7 ± 0.5	2.9 ± 0.5	2.7 ± 0.5	0.042
NT-ProBNP, pg/ml	168.7 (62.1, 436.0)	843.0 (445.5, 1265.8)	227.8 (72.3, 572.0)	< 0.001
Serum uric acid, umol/L	387.6 ± 99.3	422.3 ± 96.1	391.4 ± 99.5	0.033
Creatinine, umol/L	85.4 ± 68.1	83.2 ± 20.0	85.1 ± 64.7	0.839
eGFR, ml/min/1.73m^2^	86.5 ± 26.3	85.1 ± 30.1	86.3 ± 26.7	0.757
Medications				
Antiplatelet, n (%)	26 (7.5)	3 (7.0)	29 (7.4)	1.000
Anticoagulant, n (%)	27 (7.8)	3 (7.0)	30 (7.7)	1.000
beta-blocker, n (%)	166 (47.7)	20 (46.5)	186 (47.6)	0.883
ACEI/ARB/ARNI, n (%)	44 (10.0)	2 (4.7)	46 (11.8)	0.125
MRA, n (%)	17 (3.9)	8 (18.6)	25 (6.4)	0.002
AADs, n (%)	90 (25.9)	5 (11.6)	95 (24.3)	0.040
Statins, n (%)	62 (17.8)	7 (16.3)	69 (17.6)	0.803
Diuretics, n (%)	2 (0.6)	0 (0)	2 (0.5)	1.000
TTE				
LAD, mm	36.3 ± 5.8	44.2 ± 6.6	37.1 ± 6.4	< 0.001
LVEDD, mm	46.3 ± 4.8	49.1 ± 6.2	46.6 ± 5.0	< 0.001
LVEF, %	63.5 ± 7.0	56.7 ± 11.1	62.7 ± 7.8	< 0.001
MR	48 (13.8)	17 (39.5)	14 (35.5)	< 0.001
Mild MR	27 (7.8)	10 (23.3)	7 (64.5)	0.003
Moderate MR	21 (6.0)	7 (16.3)	7 (64.5)	0.032

LAA, left atrial appendage; TM, thrombogenic milieu; BMI, body mass index; AF, atrial fibrillation; NT-ProBNP, N-terminal pro-B type natriuretic peptide; eGFR, estimated glomerular filtration rate; ACEI, angiotensin converting enzyme inhibitor; ARB, angiotensin receptor blocker; ARNI, angiotensin receptor neprilysin inhibitor; MRA, mineralocorticoid receptor antagonist; AADs, anti-arrhythmic drugs; TTE, transthoracic echocardiography; LAD, left atrial diameter; LVEDD, left ventricular end-diastolic diameter; LVEF, left ventricular ejection fraction; MR, mitral regurgitation.

**Table 2:** Number of patients with LAAT, sludge, SEC, and TM

	LAAT	LAAT + SEC	LAA sludge	LAA SEC	LAA TM
Low TE risk, n (%)	2 (0.5)	2 (0.5)	0 (0)	6 (1.5)	10 (2.6)
Moderate TE risk, n (%)	3 (0.8)	2 (0.5)	3 (0.8)	25 (6.4)	33 (8.4)
Total, n (%)	5 (1.3)	4 (1.0)	3 (0.8)	31 (7.9)	43 (11.0)

LAA, left atrial appendage; LAAT, left atrial appendage thrombus; SEC, spontaneous echo contrast; TM, thrombogenic milieu; TE, thromboembolism.

### Predictors of patients with LAA TM

In univariate analysis, factors significantly associated with an increased risk for the presence of LAA TM were higher age (OR 1.046; 95% CI 1.005–1.089, p = 0.026), non-paroxysmal AF (OR 9.341; 95% CI 4.195–20.799, p < 0.001), congestive heart failure (OR 5.470; 95% CI 2.484–12.043, p < 0.001), a higher CHA_2_DS_2_-VASc score (OR 1.684; 95% CI 1.060–2.677, p = 0.027), a larger LAD (OR 1.202; 95% CI 1.135–1.273, p < 0.001), and MR (OR 4.087; 95% CI 2.064–8.091, p < 0.001). After combining these individual risk factors in a multivariate regression model, only non-paroxysmal AF (OR 3.121; 95% CI 1.205–8.083, p = 0.019), and a larger LAD (OR 1.134; 95% CI 1.060–1.213, p < 0.001) remained significantly associated with the presence of LAA TM. (Table 3)

**Fig. 4:**
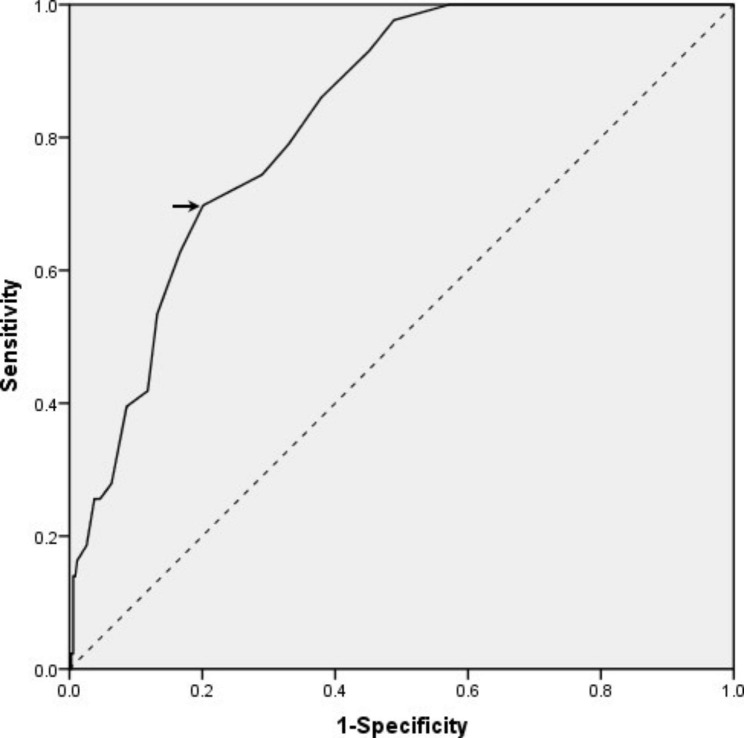
Receiver operating characteristic curve of left atrial diameter (LAD) for predicting left atrial appendage thrombogenic milieu. Arrow shows the optimal cut-off value for LAD.

As was shown in the ROC curve, the optimal cut-off value for LAD displaying the best predictive value was 40.5 mm (sensitivity = 69.8% and specificity = 79.9%; area under the curve = 0.829; Fig. 4). In addition, non-paroxysmal AF increased more than twofold risk for the presence of LAA TM.

**Table 3:** Univariate and multivariate analysis of LAA TM

Variables	Univariate analysis			Multivariate analysis	
	OR (95% CI)	P value		OR (95% CI)	P value
Age	1.046 (1.005–1.089)	0.026		1.048 (0.996–1.104)	0.071
Male sex	1.087 (0.552–2.140)	0.809			
BMI	1.032 (0.939–1.133)	0.516			
Non-paroxysmal AF	9.341 (4.195–20.799)	< 0.001		3.121 (1.205–8.083)	0.019
Hypertension	1.031 (0.530–2.005)	0.929			
Diabetes mellitus	3.187 (0.813–12.503)	0.096			
Congestive heart failure	5.470 (2.484–12.043)	< 0.001		1.894 (0.701–5.122)	0.208
Coronary artery disease	1.520 (0.498–4.639)	0.462			
CHA_2_DS_2_-VASc score	1.684 (1.060–2.677)	0.027		1.203 (0.663–2.180)	0.543
Antiplatelet	0.929 (0.269–3.208)	0.907			
Anticoagulant	0.892 (0.259–3.073)	0.856			
Hematocrit	1.035 (0.997–1.074)	0.072			
D-dimer	1.027 (0.039–26.875)	0.987			
fibrinogen	1.712 (0.968–3.027)	0.065			
LAD	1.202 (1.135–1.273)	< 0.001		1.134 (1.060–1.213)	< 0.001
MR	4.087 (2.064–8.091)	< 0.001		1.330 (0.596–2.970)	0.486

LAA, left atrial appendage; TM, thrombogenic milieu; OR, odds ratio; CI, confidence interval; BMI, body mass index; AF, atrial fibrillation; LAD, left atrial diameter; MR, mitral regurgitation.

### Management of patients with LAA TM

All patients with LAAT or sludge were prescribed with oral anticoagulant (OAC) at discharge, among whom, 7 patients received standard dose of non-Vitamin K antagonist oral anticoagulant (NOAC), 2 patients received reduced dose of NOAC, and 3 patients received Vitamin K antagonist (VKA) (target INR 2.0–3.0). After a mean duration of 117.5 ± 20.0 days, all of the thrombi and sludges resolved with a detailed recheck of TEE (Table 4). In patients with solely SEC, 22 patients underwent catheter ablation and 1 patient underwent surgical LAA ligation. No peri-operational TE event occurred. The remaining 8 patients received medication of OAC.

**Fig. 5:**
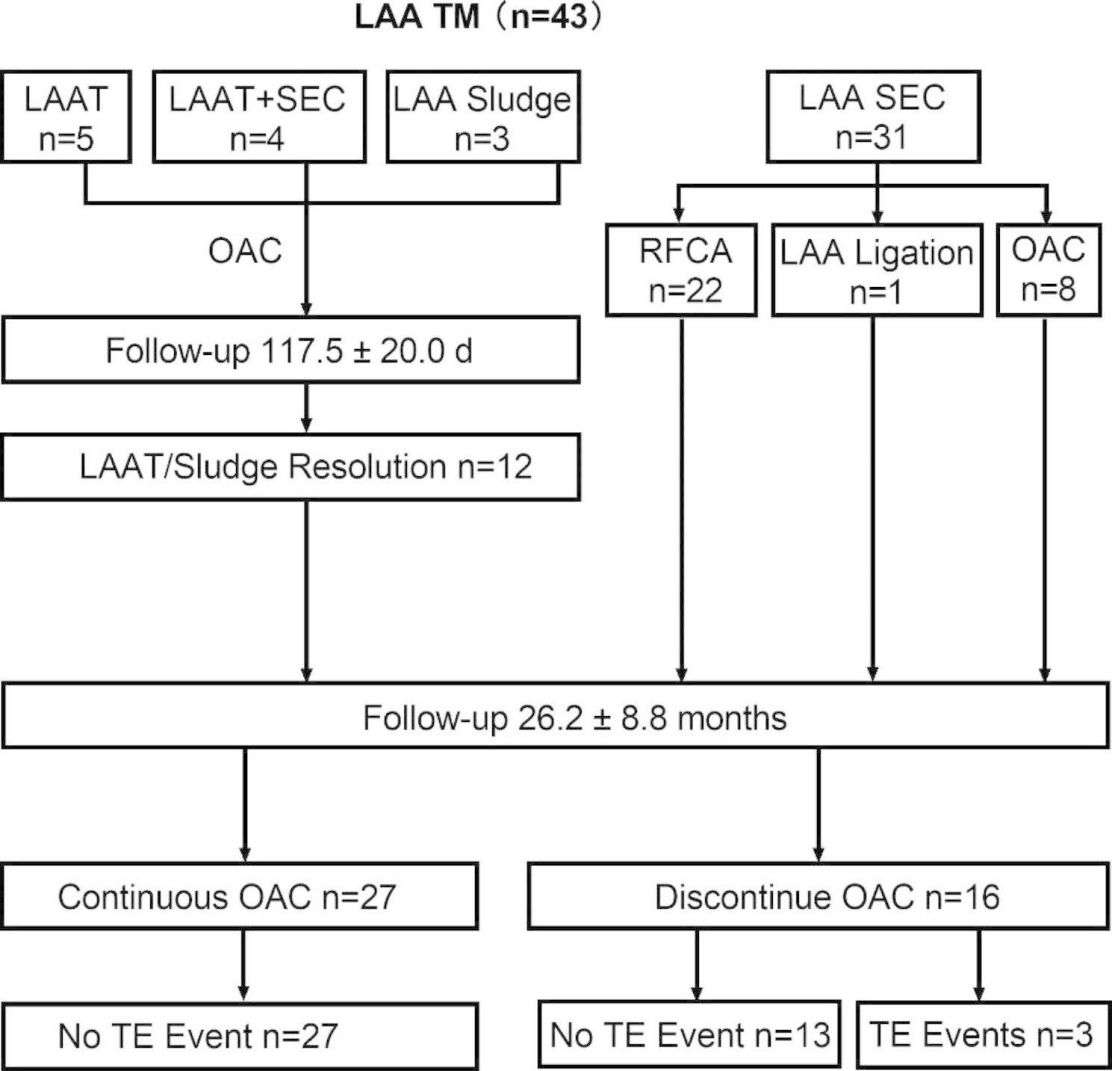
The management of patients with left atrial appendage thrombogenic milieu. LAA, left atrial appendage; TM, thrombogenic milieu; LAAT, left atrial appendage thrombosis; SEC, spontaneous echo contrast; OAC, oral anticoagulant; RFCA, radiofrequency catheter ablation; TE, thromboembolism

Over a mean follow-up of 26.2 ± 8.8 months, 27 patients (62.8%) were continuous on OAC therapy (14 patients with standard dose of NOAC, 11 patients with reduced dose of NOAC, and 2 patients with VKA). No TE events occurred in patients with continuous OAC, while 2 patients (12.5%) experienced ischemic stroke and 1 patient (6.3%) experienced peripheral artery embolism in patients discontinuing OAC. (Fig. 5)

**Table 4:** Management of patients with LAAT or sludge

Patient #	Age (y/o)	Sex	AF type	CHA_2_DS_2_-VASc score	LAD (mm)	MR	OAC before TEE	LAAT or sludge	LAA SEC	OAC after TEE	LAAT/sludge resolution	Time to resolution (day)
1	61	Male	PeAF	1	57	None	No	LAAT	No	Rivaroxaban 10 mg QD	Yes	141
2	55	Male	PeAF	0	48	None	No	LAAT	No	Rivaroxaban 20 mg QD	Yes	106
3	60	Female	PeAF	1	49	None	No	LAAT	Yes	Dabigatran 110 mg BID	Yes	94
4	48	Male	PeAF	1	48	Moderate	No	LAAT	Yes	VKA (TTR 75%)	Yes	168
5	59	Male	PeAF	1	45	None	No	LAAT	Yes	VKA (TTR 60%)	Yes	84
6	64	Female	PeAF	2	44	Mild	No	LAAT	No	Rivaroxaban 20 mg QD	Yes	140
7	63	Male	PAF	0	42	Mild	Yes	LAAT	No	Rivaroxaban 20 mg QD	Yes	135
8	59	Male	PAF	0	35	None	No	LAAT	Yes	Rivaroxaban 20 mg QD	Yes	102
9	45	Male	PeAF	1	45	None	No	LAAT	No	Rivaroxaban 20 mg QD	Yes	119
10	58	Male	PeAF	1	43	Mild	No	sludge	No	VKA (TTR 67%)	Yes	96
11	58	Male	PAF	1	38	None	No	sludge	No	Rivaroxaban 20 mg QD	Yes	121
12	55	Female	PeAF	2	45	Mild	No	sludge	No	Rivaroxaban 20 mg QD	Yes	104

LAAT, left atrial appendage thrombus; AF, atrial fibrillation; LAD, left atrial diameter; MR, mitral regurgitation; OAC, oral anticoagulant; TEE, transesophageal echocardiography; SEC, spontaneous echo contrast; PAF, paroxysmal atrial fibrillation; PeAF, persistent atrial fibrillation; VKA, Vitamin K antagonist; TTR, time in therapeutic range.

## Discussion

The major findings of our study are: (1) the prevalence of LAA TM was 11.0% in a hospital-based cohort with low to moderate TE risk; (2) non-paroxysmal AF and LAD were independently associated with the presence of LAA TM; (3) All of the LAATs or sludges could effectively resolve in 3–4 months; (4) in patients with LAA TM yet discontinuing OAC, 18.8% individuals experienced TE events. The implication is that the TE risk should not be overlooked despite a low CHA_2_DS_2_-VASc score, especially in patients with non-paroxysmal AF and enlarged LAD, and long-term OAC may be warranted to minimize the TE risk.

Evaluating TE risk of individual NVAF patients is of crucial value for decision making to long-term OAC treatment. Although the widely accepted CHADS_2_ and CHA_2_DS_2_-VASc scoring system could provide predictive value in TE risk stratification, studies have shown that moderate to severe SEC and even LAAT were not uncommon in low risk patients who underwent TEE examination. However, the prevalence of LAA TM in these patients varied from 0.6 to 29.0% according to previous studies [8, 9, 12–14]. Puwanant S et al. [12] observed LAAT, sludge or SEC in 29.0% of pre-ablation patients with CHADS_2_ score of 0 or 1. Kleemann T et al. [13] identified LAAT or dense SEC in 10.1% patients with CHADS_2_ score of 0 or 1. Yarmohammadi H et al. [8] reported that 2.3% of patients with low TE risk were detected with LAAT or sludge in an adequate coagulated cohort who were scheduled for cardioversion. Whereas a recent study by Göldi T et al. [14] shown that the prevalence of LAA TM was only 0.6% in pre-ablation patients with CHA_2_DS_2_-VASc score of 0 or 1. In the present study, we found that LAA TM could be detected in 11.0% (LAAT 2.3%, LAA sludge 0.8%, solely LAA Sect. 7.9%) of NVAF patients with low to moderate TE risk, which is comparable to Kleemann T’s finding. We believe that the variability among studies results from difference in definition of LAA TM, patient characteristics and sample size.

Several clinical features have been proposed to predict the presence of LAA TM. Studies showed that CHADS_2_ or CHA_2_DS_2_-VASc scores correlated with the risk of LAA TM [8, 14, 15]. In addition, non-paroxysmal AF has been shown to be independently associated with the presence of LAA TM [14]. In concordance with that, non-paroxysmal AF independently increased more than twofold risk for LAA TM in our study. Furthermore, certain echocardiographic parameters, such as enlarged LAD, impaired LVEF and reduced LAA flow velocity, have been revealed to predict the presence of LAA TM by several studies [8, 12, 13, 16, 17]. The role left atrium (LA) enlargement playing in TE events has been controversial. Although previous studies showed that enlarged LAD in TTE was not predictive of stroke event in two large cohorts [18, 19], echocardiographic studies reproducibly demonstrated that LA enlargement was a strong predictor of LAA TM [9, 13, 17]. We found that LAD independently predicted the presence of LAA TM after adjusting for potential confounders, with the best cut-off value of 40.5 mm. Interestingly, the medications of the present cohort differed between patients with and without LAA TM. More patients with LAA TM had medication of MRA, which might be due to a higher prevalence of congestive heart failure. In contrast, less patients with LAA TM had medication of AADs, which might be explained by a higher prevalence of non-paroxysmal AF who were more likely to accept the rate control strategy.

Short-term OAC, including NOACs and VKA, has long been used to resolve LAAT or sludge in patients with NVAF. Previous studies investigating the efficacy of NOACs or AVK showed conflicting results with the resolution rate of LAAT ranging from 41.5–85.7% [14, 20–24]. However, in the present study, all LAATs or sludges effectively resolved after OAC medication of 3–4 months, whether standard-dose NOAC, reduced-dose NOAC or VKA (TTR ranging from 60 to 75%). We believe the high resolution rate in our study may result from the Asian race and low CHA_2_DS_2_-VASc scores. Previous study demonstrated that the patients with LAAT resolution had lower CHA_2_DS_2_-VASc scores [22]. In addition, the overall resolution rate is obviously higher in the Asian race [22–24]. The racial disparity of effectiveness and safety with OAC has been discussed in various studies. A recent meta-analysis showed that Asian race was associated with lower stroke and systematic embolism rate on standard-dose NOAC therapy [25]. On the other hand, Asians were reported to be more susceptible to OAC related bleeding events [26, 27]. This phenomenon was attributed to greater platelet reactivity and endogenous fibrinolysis in Asian population [28].

The presence of LAAT or SEC was demonstrated to be associated with increased long-term TE risk and even all-cause mortality [9, 10]. However, previous studies showed that long-term OAC may compensate these adverse effects [13, 29]. Vinereanu D et al. [29] investigated the relationship between echocardiographic risk factors and clinical outcomes in a sufficiently anticoagulated cohort and found that these factors could not predict the long-term TE events. Indeed, no patients receiving continuous OAC experienced TE events in the present study, which implies the necessity of long-term coagulation in those with LAA TM, even with low CHA_2_DS_2_-VASc scores.

The present study has several limitations. Firstly, this study is a retrospective observational single-center in-hospital study with a limited sample size, which may limit the generalization of the results. Secondly, the anticoagulation rate before TEE in the study is low. Thirdly, the LAA flow velocity and grade of SEC were not available in this study, which limits the quantitative and semi-quantitative analysis of LAA TM. However, the inter- and intra-observer variability of qualitative TEE data is very low. Fourthly, only 3 patients experienced TE events in the follow-up period, which limits the multivariate analysis to evaluate the hazard ratio of discontinuing OAC to long-term TE risk. Further research based on a larger sample size is needed to investigate this issue.

## Conclusion

Although with low to moderate TE risk, the LAA TM could be identified in 11.0% in an in-hospital cohort, especially in those with non-paroxysmal AF and enlarged LAD. Short-term OAC medication could effectively resolve the LAAT or sludge. However, long-term OAC medication may still be needed to minimize the TE risk.

## Data Availability

The datasets used and/or analyzed during the current study are available from the corresponding author on reasonable request.

## List of Abbreviations

AAD: Antiarrhythmic drug
ACEI: Angiotensin converting enzyme inhibitor
AF: Atrial fibrillation
ARB: Angiotensin receptor blocker
ARNI: Angiotensin receptor neprilysin inhibitor
CI: Confidence interval
eGFR: Estimated glomerular filtration rate
INR: International normalized ratio
LAA: Left atrial appendage
LAAT: Left atrial appendage thrombus
LAA TM: Left atrial appendage thrombogenic milieu
LAD: Left atrial diameter
LVEDD: Left ventricular end-diastolic diameter
LVEF: Left ventricular ejection fraction
MR: Mitral regurgitation
MRA: Mineralocorticoid receptor antagonist
NOAC: Non-Vitamin K antagonist oral anticoagulant
NT-ProBNP: N-terminal pro-B type natriuretic peptide
NVAF: Non-valvular atrial fibrillation
OAC: Oral anticoagulant
OR: Odds ratio
PAF: Paroxysmal atrial fibrillation
PeAF: Persistent atrial fibrillation
RFCA: Radiofrequency catheter ablation
ROC: Receiver operating characteristic
SEC: Spontaneous echo contrast
TE: Thromboembolism
TEE: Transesophageal echocardiography
TTE: Transthoracic echocardiography
TTR: Time in therapeutic range
VKA: Vitamin K antagonist

## References

[1] Benjamin EJ, Wolf PA, D’Agostino RB, Silbershatz H, Kannel WB, Levy D (1998). Impact of atrial fibrillation on the risk of death: the Framingham Heart Study. Circulation.

[2] Conen D, Chae CU, Glynn RJ, Tedrow UB, Everett BM, Buring JE (2011). Risk of death and cardiovascular events in initially healthy women with new-onset atrial fibrillation. JAMA.

[3] Al-Saady NM, Obel OA, Camm AJ (1999). Left atrial appendage: structure, function, and role in thromboembolism. Heart.

[4] Beigel R, Wunderlich NC, Ho SY, Arsanjani R, Siegel RJ (2014). The left atrial appendage: anatomy, function, and noninvasive evaluation. JACC Cardiovasc Imaging.

[5] Patti G, Pengo V, Marcucci R, Cirillo P, Renda G, Santilli F (2017). The left atrial appendage: from embryology to prevention of thromboembolism. Eur Heart J.

[6] Hindricks G, Potpara T, Dagres N, Arbelo E, Bax JJ, Blomstrom-Lundqvist C (2021). 2020 ESC Guidelines for the diagnosis and management of atrial fibrillation developed in collaboration with the European Association for Cardio-Thoracic surgery (EACTS): the Task Force for the diagnosis and management of atrial fibrillation of the European Society of Cardiology (ESC) developed with the special contribution of the European Heart Rhythm Association (EHRA) of the ESC. Eur Heart J.

[7] Zabalgoitia M, Halperin JL, Pearce LA, Blackshear JL, Asinger RW, Hart RG (1998). Transesophageal echocardiographic correlates of clinical risk of thromboembolism in nonvalvular atrial fibrillation. Stroke Prevention in Atrial Fibrillation III Investigators. J Am Coll Cardiol.

[8] Yarmohammadi H, Klosterman T, Grewal G, Alraies MC, Varr BC, Lindsay B (2013). Efficacy of the CHADS(2) scoring system to assess left atrial thrombogenic milieu risk before cardioversion of non-valvular atrial fibrillation. Am J Cardiol.

[9] Lowe BS, Kusunose K, Motoki H, Varr B, Shrestha K, Whitman C (2014). Prognostic significance of left atrial appendage “sludge” in patients with atrial fibrillation: a new transesophageal echocardiographic thromboembolic risk factor. J Am Soc Echocardiogr.

[10] Bernhardt P, Schmidt H, Hammerstingl C, Luderitz B, Omran H (2005). Patients with atrial fibrillation and dense spontaneous echo contrast at high risk a prospective and serial follow-up over 12 months with transesophageal echocardiography and cerebral magnetic resonance imaging. J Am Coll Cardiol.

[11] Fatkin D, Scalia G, Jacobs N, Burstow D, Leung D, Walsh W (1996). Accuracy of biplane transesophageal echocardiography in detecting left atrial thrombus. Am J Cardiol.

[12] Puwanant S, Varr BC, Shrestha K, Hussain SK, Tang WH, Gabriel RS (2009). Role of the CHADS2 score in the evaluation of thromboembolic risk in patients with atrial fibrillation undergoing transesophageal echocardiography before pulmonary vein isolation. J Am Coll Cardiol.

[13] Kleemann T, Becker T, Strauss M, Schneider S, Seidl K (2009). Prevalence and clinical impact of left atrial thrombus and dense spontaneous echo contrast in patients with atrial fibrillation and low CHADS2 score. Eur J Echocardiogr.

[14] Goldi T, Krisai P, Knecht S, Aeschbacher S, Spies F, Zeljkovic I (2019). Prevalence and management of Atrial Thrombi in patients with Atrial Fibrillation before pulmonary vein isolation. JACC Clin Electrophysiol.

[15] Zhang E, Liu T, Li Z, Zhao J, Li G (2015). High CHA2DS2-VASc score predicts left atrial thrombus or spontaneous echo contrast detected by transesophageal echocardiography. Int J Cardiol.

[16] Balouch M, Gucuk Ipek E, Chrispin J, Bajwa RJ, Zghaib T, Berger RD (2017). Trends in Transesophageal Echocardiography Use, Findings, and clinical outcomes in the era of minimally interrupted anticoagulation for Atrial Fibrillation ablation. JACC Clin Electrophysiol.

[17] Chen J, Zhou M, Wang H, Zheng Z, Rong W, He B et al. Risk factors for left atrial thrombus or spontaneous echo contrast in non-valvular atrial fibrillation patients with low CHA2DS2-VASc score. J Thromb Thrombolysis. 2021:Epub ahead of print. 10.1007/s11239-021-02554-9 34476733

[18] Olshansky B, Heller EN, Mitchell LB, Chandler M, Slater W, Green M (2005). Are transthoracic echocardiographic parameters associated with atrial fibrillation recurrence or stroke? Results from the Atrial Fibrillation Follow-Up investigation of Rhythm Management (AFFIRM) study. J Am Coll Cardiol.

[19] Broughton ST, O’Neal WT, Salahuddin T, Soliman EZ (2016). The influence of Left Atrial Enlargement on the relationship between Atrial Fibrillation and Stroke. J Stroke Cerebrovasc Dis.

[20] Lip GY, Hammerstingl C, Marin F, Cappato R, Meng IL, Kirsch B (2016). Left atrial thrombus resolution in atrial fibrillation or flutter: results of a prospective study with rivaroxaban (X-TRA) and a retrospective observational registry providing baseline data (CLOT-AF). Am Heart J.

[21] Da Costa A, Delolme C, Guichard JB, Gerbay A, Pierrard R, Romeyer-Bouchard C (2017). Comparison of prevalence and management of left atrial appendage thrombi under old and new anticoagulants prior to left atrial catheter ablation. Am Heart J.

[22] Yang Y, Du X, Dong J, Ma C (2019). Outcome of anticoagulation therapy of left atrial Thrombus or sludge in patients with Nonvalvular Atrial Fibrillation or Flutter. Am J Med Sci.

[23] Lin C, Quan J, Bao Y, Hua W, Ke M, Zhang N (2020). Outcome of non-vitamin K oral anticoagulants in the treatment of left atrial/left atrial appendage thrombus in patients with nonvalvular atrial fibrillation. J Cardiovasc Electrophysiol.

[24] Feng M, Lin H, He B, Wang B, Chen X, Chu H (2021). The Safety and Efficacy of Standard-Dose versus Low-Dose Non-Vitamin K antagonist oral anticoagulants in patients with nonvalvular atrial fibrillation and left atrial appendage Thrombus. J Healthc Eng.

[25] Wang KL, Lip GY, Lin SJ, Chiang CE, Non-Vitamin K (2015). Antagonist oral anticoagulants for Stroke Prevention in Asian Patients with Nonvalvular Atrial Fibrillation: Meta-Analysis. Stroke.

[26] Shen AY, Yao JF, Brar SS, Jorgensen MB, Chen W (2007). Racial/ethnic differences in the risk of intracranial hemorrhage among patients with atrial fibrillation. J Am Coll Cardiol.

[27] Chao TF, Chen SA, Ruff CT, Hamershock RA, Mercuri MF, Antman EM (2019). Clinical outcomes, edoxaban concentration, and anti-factor xa activity of asian patients with atrial fibrillation compared with non-Asians in the ENGAGE AF-TIMI 48 trial. Eur Heart J.

[28] Gue YX, Inoue N, Spinthakis N, Takei A, Takahara H, Otsui K (2019). Thrombotic Profile and oral anticoagulation in asian and non-asian patients with Nonvalvular Atrial Fibrillation. J Am Coll Cardiol.

[29] Vinereanu D, Lopes RD, Mulder H, Gersh BJ, Hanna M, de Barros ESPGM (2017). Echocardiographic risk factors for stroke and outcomes in patients with Atrial Fibrillation Anticoagulated with Apixaban or Warfarin. Stroke.

